# A Flowering Morphological Investigation, Fruit Fatty Acids, and Mineral Elements Dynamic Changes of *Idesia polycarpa* Maxim

**DOI:** 10.3390/plants13182663

**Published:** 2024-09-23

**Authors:** Yanpeng Wang, Cuiyu Liu, Jiasong Hu, Kaiyun Wu, Bangchu Gong, Yang Xu

**Affiliations:** 1 Research Institute of Subtropical Forestry, Chinese Academy of Forestry, Hangzhou 311400, China; yanpengwang@caf.ac.cn (Y.W.); ankar_liu@163.com (C.L.); wukaiyun99@163.com (K.W.); gbc666@sina.cn (B.G.); 2 Guizhou Forestry and Grassland Development Co., Ltd., Guiyang 550000, China; notohujiasong@163.com

**Keywords:** *Idesia polycarpa*, bisexual flower, fatty acids, oil production, morphology

## Abstract

*Idesia polycarpa* Maxim is a high-value species of fruit oil with edible, abundant linoleic acid and polyphenols. *Idesia polycarpa* is described as a dioecious species, and the flowers are male; female and bisexual flowers are produced on separate plants. In order to explore the flower types of *Idesia polycarpa*, the morphology of its flowers and inflorescence were investigated in this study. The flower and inflorescence types, the diameter, and the flowering sequencing in male and female inflorescence were determined. We also detected the length, width, and fresh weight of leaves, shoots, and female inflorescence, as well as the length and fresh weight of the petiole during the development. Additionally, we compared the length, width, the length/width ratio, and the flowering density between 5- and 7-year-old female trees. The phenological period observation of *Idesia polycarpa* showed that the development process can be roughly divided into 12 stages, including bud burst, leaf expansion, inflorescence growth, initial flowering, full flowering, flower decline, initial fruiting, fruit enlargement, fruit color change, fruit ripening, post-ripening of fruit, and leaf fall periods. Furthermore, four elites’ fruit determined the oil content and the composition of fatty acid content during the development. The dynamic of fatty acids contents, the palrnitic acid, palmitoleic acid, stearic acid, oleic acid, and linolenic acid contents were detected during the fruit development of four elites. Moreover, the mineral elements content of fruit of four elites during development were determined. The patterns of vegetative and reproductive growth in young dioecious trees of *Idesia polycarpa* provided the theoretical basis for artificial pruning and training.

## 1. Introduction

*Idesia polycarpa* is a member of Salicaceae family and is native to eastern Asia, widely distributed in China, Korea, and Japan [1,2]. *Idesia polycarpa* is well known for its edible fruit oil, hygienical and medical functions, and biodiesel raw material [3,4,5]. In China, *Idesia polycarpa* is mainly distributed south of the Qinling Mountains and the Huaihe River and is famous for its high oil production and high lipid content [6]. Besides oil production, morphological traits, such as leaves, flowers, and fruit deeply influenced the overall physiology, yield, and quality of oil for *Idesia polycarpa*. Furthermore, the leaves were proved to have hemostatic roles, seed extractions were used as an insecticide, and the fruit extractions have anti-obesity properties [6,7].

*Idesia polycarpa* is described as a dioecious tree species. It is difficult to distinguish the sex of the plants at the seedling stage due to most plants flowering for at least 4 or 5 years before reaching reproductive maturity after planting [2,8]. In recent years, individual *Idesia polycarpa* with bisexual flowers were found [8]. However, only male flowers or bisexual flowers exist, the ovaries develop normally and produce fruits, and then oil is produced. Therefore, it is very important for us to explore the characteristics of flowering, fruiting, and floral initiation of the *Idesia polycarpa* species. The sex of plants is more complicated than that of animals. Even though there is no consensus on the classification of plant sex types, the sex of plants is generally classified from the solitary flower, plants, and population levels.

For plant growth and development, the annual growth is an important indicator for young trees (3 years old) of *Idesia polycarpa*. A previous study has shown that the pattern of vegetative growth in young *Idesia polycarpa* trees was different in the bud growth, leaf, and canopy size [9]. The phenology of the leaves and flowers is very important evidence for the sexual specialization and oil production. The study of branching, flowering, and the fruit production phenomenon between male and female plants provided a basis theory for sexual selection, fertilizer supply, and flowering control [8,9,10].

With the rapid growth in demand for edible oil, *Idesia polycarpa* has gradually attracted the attention of researchers due to its high lipid, oil yield and nutritive values [11]. *Idesia polycarpa* is a high oil production woody oil plant, with seeds containing 26.26% oil on a dry basis and the pulp containing 43.6% oil, resulting in an oil output of 2.25–3.75 tons per hectare [6]. *Idesia polycarpa* pulp oil was abundant in unsaturated fatty acids and active substances and possessed a certain free radical scavenging ability [12]. Both pulp and seed can be extracted for high-quality oil, and the oil contains relatively high linoleic acids, which accounts for 66–81% of the total fatty acids [3]. However, previous studies concentrated on the fruit oil extraction technology and modification [13,14], and only a few studies have investigated the chemical properties, bioactive ingredients, and free radical scavenging capacity of pulp oil [12]. The dynamically changing total oil content during fruit development has been seldom studied. Furthermore, the dynamic accumulation patterns and contents of palrnitic acid, palmitoleic acid, stearic acid, oleic acid, and linolenic acid in different elites of *Idesia polycarpa* have not been systematically compared and analyzed.

Nutrients serve as the fundamental building blocks for the growth and development of plants. Carbon constitutes the basic structure of cells, while nitrogen, phosphorus, and potassium play crucial roles in the nutrient exchange between plants and their surrounding environment [15]. The nutrient content in leaves and fruits indicates the plant growth status and habitat conditions and reflects the nutrient conditions of fruit and soil to some extent [16]. In addition, knowing the dynamic of mineral content in fruit is important for quality improvement and guiding fertilization.

Previously, studies have focused on the sexual identification, root morphology, and soil microorganisms of *Idesia polycarpa*. However, the morphology of leaves, flowers, and fruit were seldom studied. Furthermore, the dynamic accumulation of fatty acid oils and their composition and the mineral contents changes in fruit were also not researched thoroughly. Therefore, the main purpose of this study was to investigate the floral morphology characteristics and types of male, female, and bisexual flowers, and the development dynamics of the leaves and fruit of *Idesia polycarpa*. We also determined the phenology of the leaves, flowers, and fruit. Moreover, the oil content and its composition as well as the mineral content changes of *Idesia polycarpa* fruit were examined in different species. Our study of *Idesia polycarpa* lays the foundation for a better understanding of its flowering, fruiting, and oil production.

## 2. Materials and Methods

### 2.1. Plant Material

Our study was conducted in Guiding, Guizhou province, China (107°08′–107°15′ E, and 26°40′–26°47′ N). The climate of Guiding county belongs to the subtropical monsoon humid climate. The annual average temperature is 15.5 °C, the annual cumulative sunshine is 1073.9 h, the frost-free period is 289 days, the annual average precipitation is 1084.8 mm, and the average relative humidity is 78%. All the materials were collected in the same orchard in Guiding county. The *Idesia polycarpa* trees were planted in 2019, and the leaf, shoot, flower, inflorescence and fruit materials were picked from five-year-old flowering trees. Female and male trees were labeled and checked for two consecutive years to ensure gender accuracy. In total, 100 male and female trees of each were investigated, respectively, to confirm the sex characteristics of *Idesia polycarpa*.

### 2.2. Measurement of Development Parameters of Idesia polycarpa

A total of 100 flowers, 50 shoots, and 50 inflorescences from each flowering tree were picked and detected for the flower index. At least 100 male and female trees with relatively uniform height and consistent trunk thickness and healthy male and female flowering trees were chosen for the investigation (Table 1). We detected the flower characteristics of *Idesia polycarpa*, such as flower types, the diameters of different types of flowers, and the diameter of male/female flowers during development with a digital caliper (IP54, GB/T 21389-2008 [17], http://www.chinachengliang.com, accessed on 10 May 2023). In addition, the length, width, and single fresh weight of 200 leaves, the length and fresh weight of 200 petioles, and the length, diameter, and fresh weight of 200 shoots were measured during the growing periods from March to October (Table 1). Furthermore, 50 infructescences of female trees were picked for the determination of the length, width and single fresh weight. Moreover, 100 fruits were taken from each inflorescence (three replicates) for the determination of the transverse and longitudinal diameter (Table 1). In total, 100 5- and 7-year-old trees, respectively, were measured for inflorescence types, the length of inflorescence, the width of inflorescence, the length/width ratio of inflorescence, and the flower density of female and male trees during flowering (Table 1).

In addition to the above, the phenological period of *Idesia polycarpa* was investigated, including the periods of bud burst, leaf expansion, beginning of inflorescence growth, initial flowering, full flowering, flower decline, initial fruiting, fruit enlargement, fruit color change, fruit ripening, post-ripening, and leaf fall from March to October during the development of *Idesia polycarpa* (Table 1).

For the fatty acids and mineral elements determination, the fruit of QC123, QC49, QC6, and DY22, the four selected elites of *Idesia polycarpa*, was measured (Table 1). The fruit fatty acids and mineral contents were picked and detected on 0, 10, 25, 40, 55, 70, 85, 100, 115, 130, 145, 160, and 175 days after flowering (DAF). The fresh weight and dry weight of single fruit for QC123, QC49, QC6, and DY22 were 0.35 ± 0.02 g and 0.18 ± 0.01 g, 0.31 ± 0.02 g and 0.16 ± 0.01 g, 0.59 ± 0.01 g and 0.29 ± 0.01 g, and 0.66 ± 0.03 g and 0.34 ± 0.01 g at maturity. The total fatty acid oil contents of QC123, QC49, QC6, and DY22 were 32.2 ± 1.00%, 25.23 ± 1.00%, 31.86 ± 1.67%, and 19 ± 0.16%.

### 2.3. The Fatty Acid and Composition Contents Determination

All samples were picked and preliminarily screened from 5-year-old trees from a local orchard at the maturity stage of fruit (2 kg). Uniform size, ripeness, and no rotten fruit were chosen randomly from four directions from three trees with triple repetition of each dominant plant, and the fruit was immediately frozen in liquid nitrogen and stored at −80 °C in an ultra-low temperature freezer for further analysis. The *Idesia polycarpa* fruit was prepared according to the methods described by Zhang [12]. A total of 0.5 kg of fruit of different superior stains of *Idesia polycarpa* was used in this study. The oil content of *Idesia polycarpa* fruit was determined as in a previous study described by AOCS Am 2-93 [18], and the fatty acids composition was determined based on the methods described by Shi and Zhang’s as follows [12,19]. The Soxhlet method was used for the *Idesia polycarpa* oil extraction before esterification. Then the prepared oil samples were esterified according to the description of the ISO 5509:2000 method [20]. The quantification of the fatty acid methyl esters of oils was analyzed using 7890A gas chromatography (Agilent Technologies, Santa Clara, CA, USA) equipped with a flame ionization detector (GC-FID) and capillary column (HP-INNOWAX, 30 m × 0.25 mm × 0.25 μm). The method was described by Wu [21]: He as the carrier gas, injector temperature of 220 °C, split ratio 1:100, and detector temperature of 275 °C. The column was held for 1 min at 140 °C and then programmed at 4 °C/min to 250 °C. The external standard calibration method was used to quantify the samples.

### 2.4. Determination of Mineral Elements of Fruits during Different Development Periods

Three samples for each indicator with three replicates were used for the fruit (including pulp and seed) determination. The total nitrogen content (N) was determined with an automatic elemental analyzer (Euro Vector EA3000, Shanghai Wolong Instrument Co., Ltd., Shanghai, China). The total phosphorus (P) content and the total potassium (K) content were determined by the molybdenum antimony anticolorimetric method and the flame photometer method, respectively [22]. Each 0.2 g fruit sample was accurately weighed into a 50 mL polytetrafluoroethylene (PTFE) tube. Then, 3 mL of 65% HNO_3_ (*w*/*w*) solution and 2 mL of 30% H_2_O_2_ (*w*/*w*) solution were added to the sample in a PTFE tube. Next, the PTFE tube was digested in a MARS 5 Microwave System (CEM). The calcium (Ca), magnesium (Mg), iron (Fe), manganese (Mn), copper (Cu), and zinc (Zn) were quantified using ICP-OES (Thermo, Shimadzu, Japan).

### 2.5. Data Analysis

Each sample of oil and mineral content was determined in triplicate, and the results were presented as mean ± standard deviation (SD). Using SPSS 21.0 Statistical software (IBM SPSS Inc., Armonk, NY, USA, accessed on 10 May 2023), data were compared using Duncan’s multiple range test at *p* < 0.05. The plot used Origin 2021 (Origin Lab Inc., Northampton, MA, USA, accessed on 10 May 2023) and Adobe Illustrate CC 2018 (Adobe Systems Inc., San Jose, CA, USA, accessed on 13 May 2023).

## 3. Results

### 3.1. Flower Types of Idesia polycarpa

**Figure 1:**
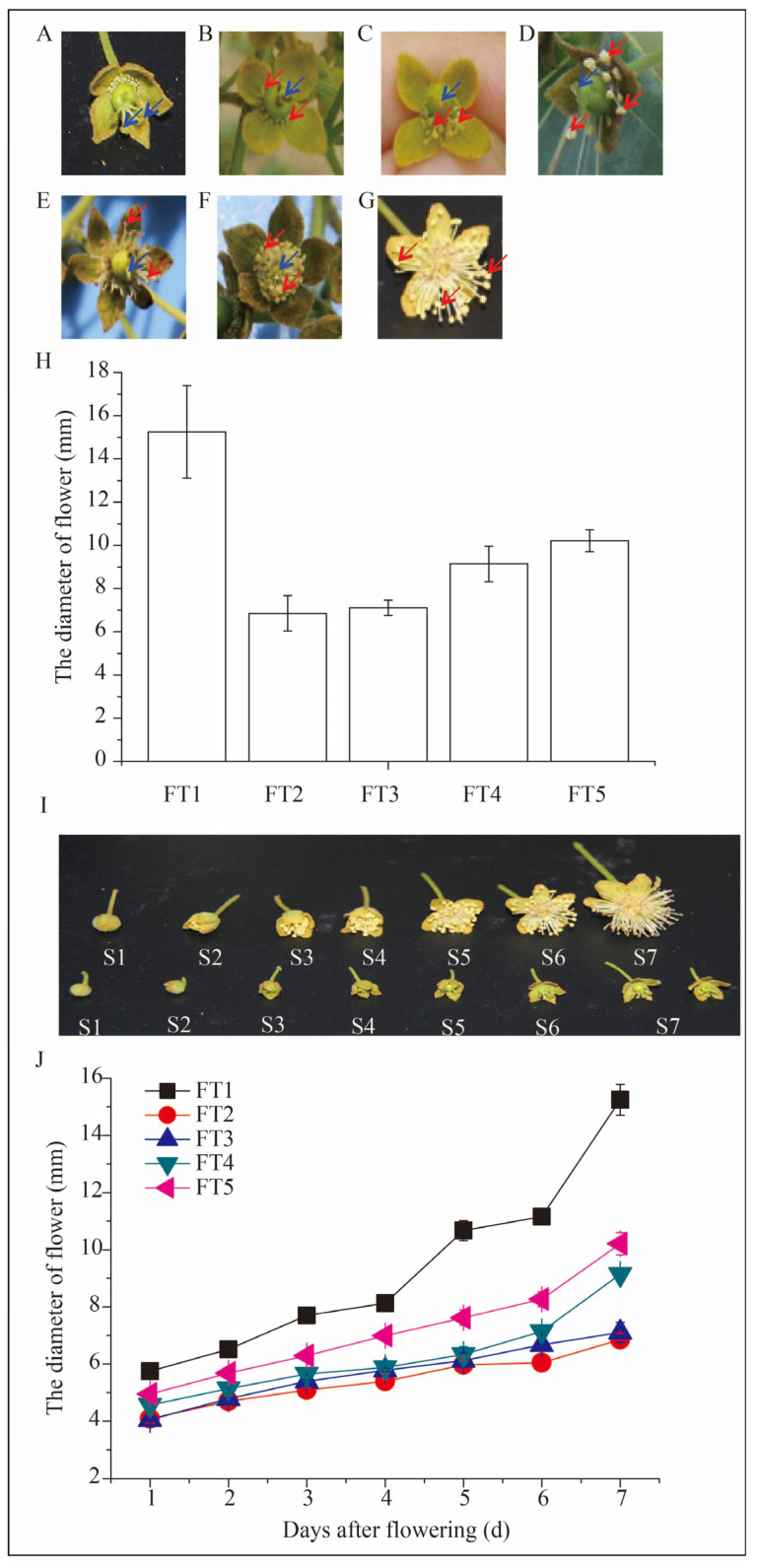
Diameter of the flower aspects of different flower types of *Idesia polycarpa*. (**A**): male flower; (**B**–**F**): bisexual flowers. Red arrow: stamen, blue arrow: pistil. (**G**): female flower; (**H**): determination of the flower diameter of different flower types during flowering. FT1: female flower; FT2: male flower; FT3–5: different types of bisexual flowers; (**I**): the morphological observation of male and female flowers during different development. Horizontal first row: male flower, horizontal second row: female flower. (**J**): diameter of different types of flowers during flowering.

The entire investigation was undertaken during the same flowering time to ensure uniformity of flowering. Our study showed that the flowers of *Idesia polycarpa*. were of at least seven types, but mainly female and male flowers (Figure 1A,G). The female flower was identified by a gynecium, which was syncarpous, and the ovary and style were together with a plicated stigma (Figure 1A). The ovary of the female flower was superior, globose, with five or six spreading outward styles and with an obovate stigma. In addition, we did not observe pollen in the antherode of the female flowers; however, many short filaments of the staminodes were observed (Figure 1A). The male flower was distinguished by an obvious andrecium, but the pistillode was small, short, and shriveled (Figure 1G). We found five types of bisexual flowers of *Idesia polycarpa*, which were as follows: (i) the flower had an ovary and style with an gynecium, and the andrecium with pollen was small and thin and on the base around the ovary (Figure 1B); (ii) the flower was almost the same as the flower in Figure 1B, but the difference was the andrecium was longer than Figure 1B (Figure 1C); (iii) the flower had an ovary and style with an gynecium, but the difference compared to B and C was that the andrecium was longer and bigger than them (Figure 1D); (iv) it was a bisexual flower with development of the pistil and stamen, which could be developed into fruit (Figure 1E); (v) both the pistil and stamen were identified, and they were covered on the ovary, but the stamen was much shorter than that in the flower in Figure 1G (Figure 1F).

The diameter of different types of flowers was also determined in this study (Figure 1H). We found that the female flowers of FL1 were significantly longer than any other types of flowers (Figure 1H). In addition, regarding the diameter, there were no significant differences among bisexual flowers of FL3, 4, and 5 (Figure 1H). Moreover, the diameter of male flowers was the smaller than other types of flowers (Figure 1A,H).

### 3.2. The Determination of Flower Diameter during Flowering

The dynamic process of flowering was investigated for male and female flowers. We recorded seven stages of flowering from bud (S1), flower opening slightly (S2–S4), and flower in full flowering (S5–S7, Figure 1J). As previously described, the female flower was bigger than the male flower (Figure 1H). The diameter of different flower types was determined during different stages (Figure 1H). Results showed that there were no significant differences between the five studied types of flowers before S2 in flower diameter. However, the female flower (FL1) was higher than any other types of flowers after the S3 during the flowering and maintained the highest diameter until S7 (Figure 1G,I,J). The flower diameter of FL5 was significantly higher than the other three types of flowers before S6, but there was no significant difference compared with FL4 at S7. The flower diameters of FL2 and FL3 were not significantly different at S7, and the female flower (FL2) was the lowest at the end of the flowering (Figure 1G,I,J).

### 3.3. The Leaf and Shoot Dynamics of Idesia polycarpa

**Figure 2:**
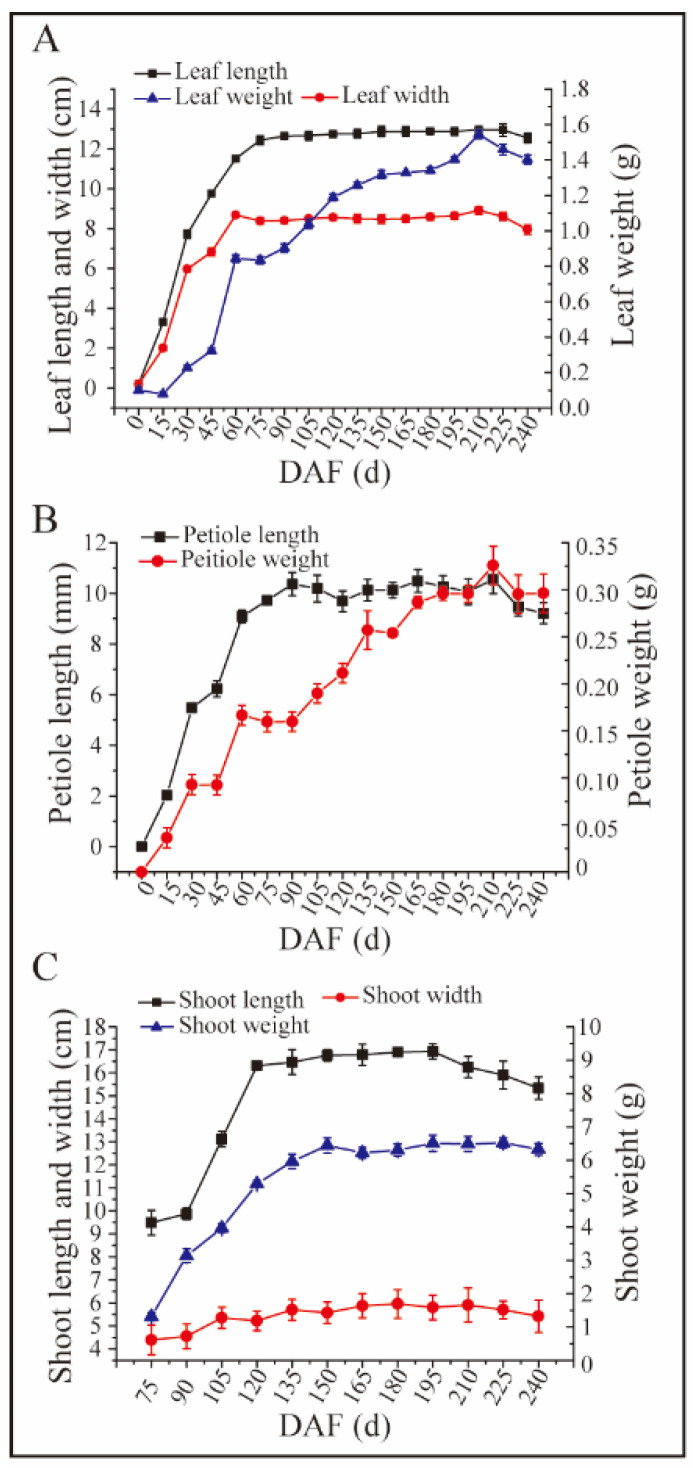
The determination of leaf, petiole, and shoot during development. (**A**): the length, width and fresh weight of leaves during the growing periods, (**B**): the length and fresh weight of the petiole during the growing periods, (**C**): the length, diameter and fresh weight of shoots during the growing periods.

The length of leaves increased quickly from 1 March to 22 June, then maintained a relatively stable length of 12 centimeters until the end of October (Figure 2A). The length of petiole showed the same growth trends during different months (Figure 2B). The width of leaves increased quickly from March to 5 May; however, the width of the leaves seems to stop increasing and maintained a relatively stable value of 8 centimeters at the end of October (Figure 2A). The changes in the fresh weight of petiole was consistent with the fresh weight of leaves (Figure 2A,B). Results showed that the fresh weight of the leaves and petioles significantly increased from March to early July, then slightly decreased during August (Figure 2A,B). However, both the fresh weight of the leaves and petioles increased again in September, then decreased in October (Figure 2A,B). In this study, the shoot length and fresh weight had three increase periods and three decrease periods, and the trends of these two indexes changed similarly (Figure 2C). Three increase periods of shoot length and fresh weight included the whole of June, 4 July to 2 August, and 17 August to 21 September, and the three decrease periods included 4 to 21 July, 2 August to 17 August, and 14 October to the end (Figure 2C). However, the diameter of the shoots changed slightly and not significantly during all the growing periods (Figure 2C).

### 3.4. Different Types of Inflorescence of Idesia polycarpa

**Figure 3:**
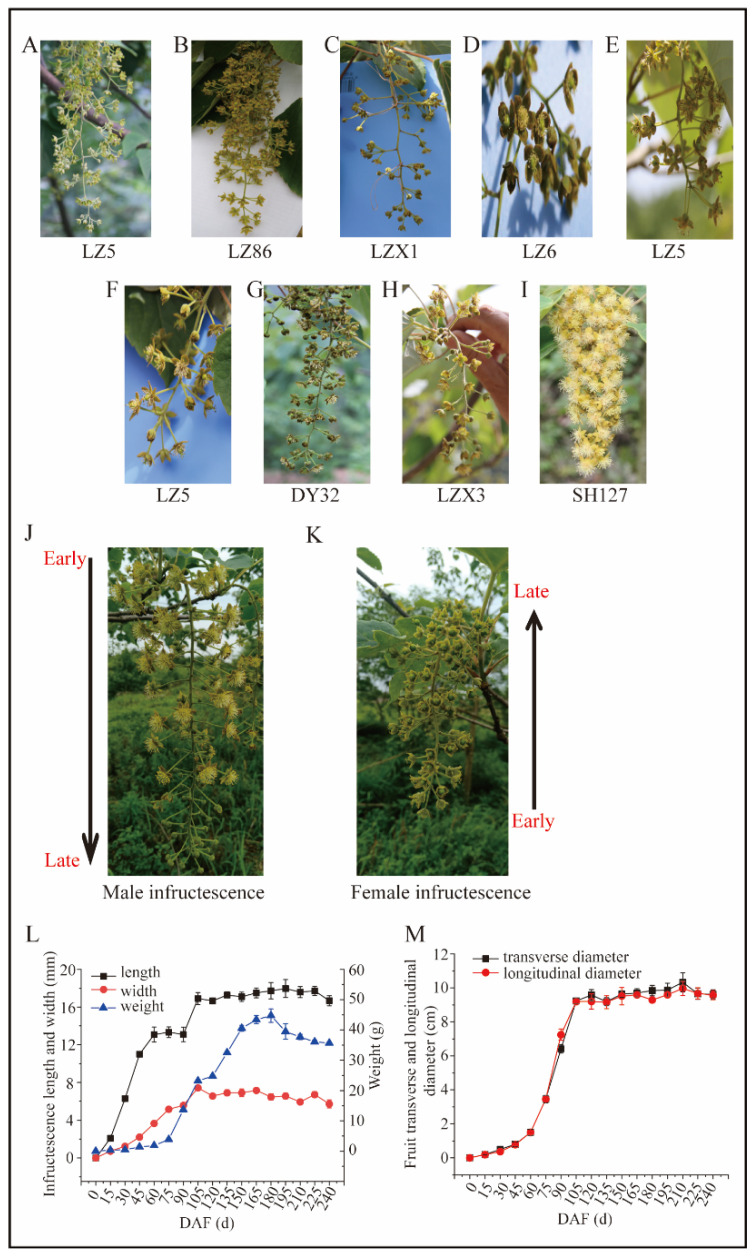
Different types of inflorescences of *Idesia polycarpa*. (**A**): the inflorescence of female flowers; (**B**): the inflorescence is mainly female flowers, with a small amount of bisexual flowers; (**C**): the inflorescence of mainly female flowers, with a small amount of male flowers and less bisexual flowers; (**D**): the inflorescence is mainly bisexual flowers, with a small amount of female and male flowers; (**E**): the inflorescence of bisexual flowers; (**F**): the inflorescence is mainly bisexual flowers, with less male flowers; (**G**): the inflorescence is half bisexual flowers and half male flowers; (**H**): the inflorescence is mainly male flowers, with less bisexual flowers; (**I**): the inflorescence of male flowers. (**J**): male inflorescence; (**K**): female inflorescence; (**L**): the length, width and fresh weight of inflorescence during development; (**M**): the transverse diameter and longitudinal diameter of fruit in different time during development.

The inflorescence of the *Idesia polycarpa* are raceme, which is an inverted cone shape. The inflorescences were mainly female and male based on our investigation, and these two types of inflorescence account for more than 95% (Figure 3A,I). In addition, the bisexual inflorescences were diverse and were of seven types (Figure 3B–H). The inflorescences of these nine types, which were composed of lateral branches and pedicels and the calyx, were colorful and without petals, which were yellow, green and gray (Figure 3). The female and male inflorescences could be easily distinguished, while the bisexual inflorescences could be divided into seven types, which were (i) mainly female flowers, with small amount of bisexual flowers (Figure 3B); (ii) mainly female flowers, with small amount of male flowers and less bisexual flowers (Figure 3C); (iii) mainly bisexual flowers, with smaller amounts of female and male flowers (Figure 3D); (iv) bisexual flowers (Figure 3E); (v) mainly bisexual flowers, with less male flowers (Figure 3F); (vi) half bisexual flowers and half male flowers (Figure 3G); (vi) mainly male flowers, with less bisexual flowers (Figure 3H); (vii) male flowers (Figure 3I). Interestingly, we did not find the existence of male and female flowers in the same inflorescence.

We also investigated the flowering order in the same inflorescence between male and female inflorescences. Results showed that the flowering order in female inflorescences was from the base to the top; on the contrary, the flowering order in male inflorescences was from the top to the base (Figure 3J,K).

### 3.5. The Growth and Development Dynamics of Infructescence and Fruit

In this study, the length, width, and the fresh weight of infructescence, as well as the fruit transverse and longitudinal diameter (Figure 3) were determined. Results showed that the length of infructescence prior to increase was significantly higher when compared with the width of infructescence from March to 22 June; then, both of them maintained a relative steady value of 18 and 6 cm, with slight changes from 22 June to the end of October (Figure 3L). The fruit transverse and longitudinal diameter increased quickly from May to 22 June, and then maintained a steady value of 9 cm until the end of October (Figure 3M). As shown in Figure 3M, we can see the trend of changes were similar between the fruit transverse and longitudinal diameters.

**Figure 4:**
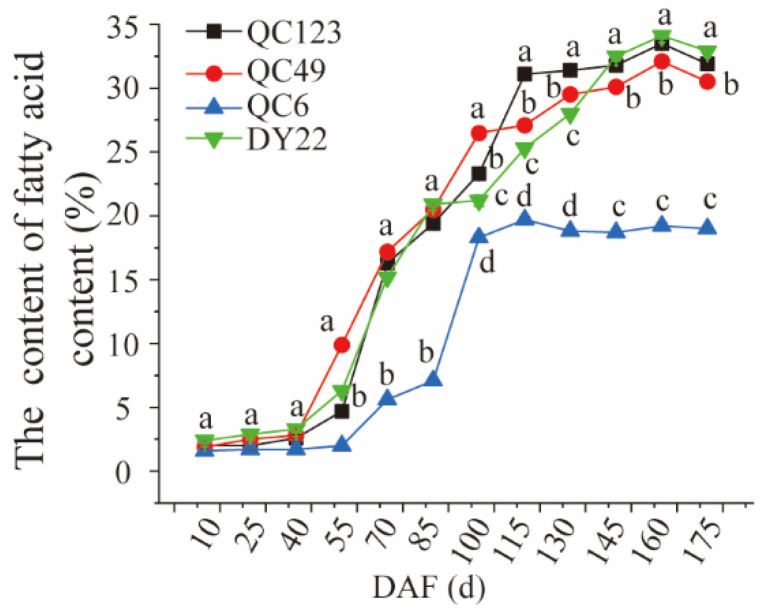
The fruit fatty acid content in fruit of four elites of *Idesia polycarpa* during development. Different letters indicate significant differences (*p* < 0.05) within the different lines.

**Figure 5:**
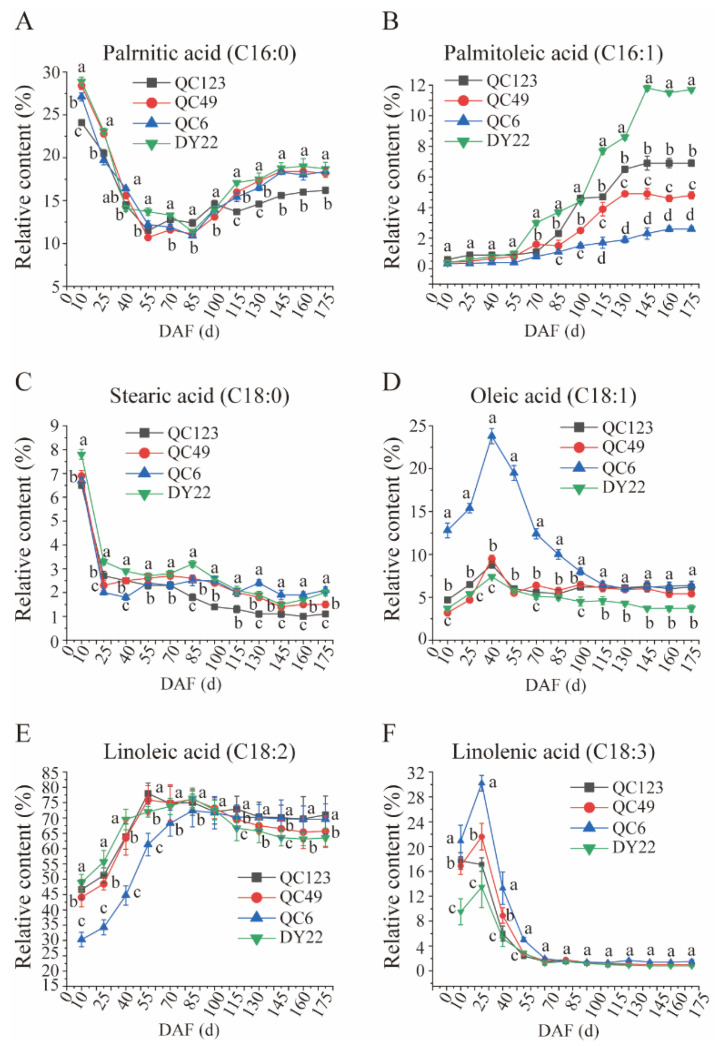
Dynamic changes in fatty acid composition contents in different *Idesia polycarpa* elites during fruit development. (**A**): Palrnitic acid (C16:0), (**B**): palmitoleic acid (C16:1), (**C**): stearic acid (C18:0), (**D**): oleic acid (C18:1), (**E**): linoleic acid (C18:2), (**F**): linolenic acid (C18:3). Different letters indicate significant differences (*p* < 0.05) within the different lines.

Similar trends in the fresh weight occurred during the growing periods between infructescence and fruit (Figure 3L,M). The fresh weight of infructescence increased during the middle of April to the beginning of August, even though the length and width of the infructescences stopped growing in this period. The fresh weight of infructescence had a top value of 18 centimeter (cm), then decreased slightly at the end of the October (Figure 3L,M). We also detected the fruit’s fatty acid content of four elites during development (Figure 4). More details were shown in Figure 5.

### 3.6. Comparison of Inflorescences in Five- and Seven-Year-Old Trees

The morphology of the male and female inflorescences in five-year-old and seven-year-old *Idesia polycarpa* was investigated (Table 2). Results showed that the length of the male inflorescences was significantly longer than that of the female inflorescences. Additionally, the width of the female inflorescences was significantly wider than that of the male inflorescences, which resulted in a significantly higher length-to-width ratio for the male inflorescences compared to the female inflorescences. Furthermore, the differences between the female and male inflorescences become more significant as the tree aged. Considering that *Idesia polycarpa* are insect flowers, wider male inflorescences and narrow female flowers had greater advantages in attracting insects for pollination and improving the pollination efficiency. Moreover, the flowering intensity of the male inflorescences was significantly higher than that of the female inflorescences during the same time. The differences in the flowering intensity between male and female inflorescences were much more obvious with the increasing age of the trees, which indicated that the flowering time of male flowers was earlier than that of female flowers. According to our study, the inflorescence of male flowers was one week earlier than female flowers in the spring.

### 3.7. The Phenological Period Investigation of Idesia polycarpa

According to the phenological period of *Idesia polycarpa* as described in Table 3, the growth and development process can be roughly divided into 12 stages, including bud burst, leaf expansion, inflorescence growth, initial flowering, full flowering, flower decline, initial fruiting, fruit enlargement, fruit color change, fruit ripening, post-ripening of fruit, and leaf fall periods (Table 3). During these different growth stages, there is some overlap in the growth requirements of leaves and flowers. Therefore, it is necessary to coordinate fertilization to consider the growth needs of both leaves and flowers. This means that the timing and method of fertilization need to be adjusted according to the specific requirements of *Idesia polycarpa* at different growth stages to ensure that the plant receives adequate nutritional support throughout the growing season.

### 3.8. The Dynamic Analysis of Fruit Fatty Acids Content during Development

We first determined the fatty acid content of elite QC123, QC4-9, QC6, and DY22 (Figure 4). Results showed that the DY22 had the highest fatty acid content, with an average value of 32.5% on 175 DAF. The second was QC123, with an average fatty acid content of 32.5% at the end of fruit development. The fatty acid content of QC4-9 was 26.5% on 175 DAF. The fatty acid content of QC6 was lower than any of the other three elites during the whole fruit development, with an average fatty acid content of 18.2% on 175 DAF. We also defined the types of fruit according to scale combined with the fatty acid content, which were small fruit with high fatty acid content (QC123), small fruit with middle fatty acid content (QC49), big fruit with high fatty acid content (DY22), and big fruit with low fatty acid content (QC6).

In this study, the contents of palmitic acid (C16:0), palmitoleic acid (C16:1), stearic acid (C18:0), oleic acid (C18:1), linoleic acid (C18:2), and linolenic acid (C18:3) were detected. The content of palrnitic acid of these four elite trees decreased during 10 to 85 DAF but increased slightly during 85 to 175 DAF during fruit development. The content of palrnitic acid of these four elites showed no significant differences before 100 DAF; however, the content of palrnitic acid in QC49, DY22, and QC6 was significantly higher than QC123 after 70 DAF (Figure 5A). The content of palmitoleic acid increased during fruit development in the four elite trees (Figure 5B). Small changes in the palmitoleic acid content were detected in the four elite trees before 85 DAF; however, all elites increased after 85 DAF. During this period, the content of palmitoleic acid in DY22 sharply increased and maintained the highest content. QC6 was the lowest one among these four elites (Figure 5B). The content of stearic acid among the four elites declined after 10 DAF and maintained a content from 1.5~2.3 during 25 to 175 DAF (Figure 5C). The content of stearic acid in QC49, DY22, and QC6 was higher than 123 during 85 to 175 DAF (Figure 5C). The content of oleic acid among these four elites increased after 10 DAF, then reached its top point at 40 DAF (Figure 5D). The content of oleic acid in QC6 was higher than QC123, QC49, and DY22 before 115 DAF and was not significant for QC123, QC49, and QC6 during 115 to 175 DAF but higher than DY22 (Figure 5D). The content of linoleic acid increased from 30 to 70, then decreased from 85 to 175 DAF, and maintained a content of 58 ~ 64 mg/kg (Figure 5E). The content of linolenic acid increased at the point of 25 DAF, then sharply decreased after that, and slightly changed from 70 to 175 with a value between 1 to 3 mg/kg (Figure 5F).

### 3.9. The Dynamic Analysis of Mineral Contents in Fruit during Development

**Figure 6:**
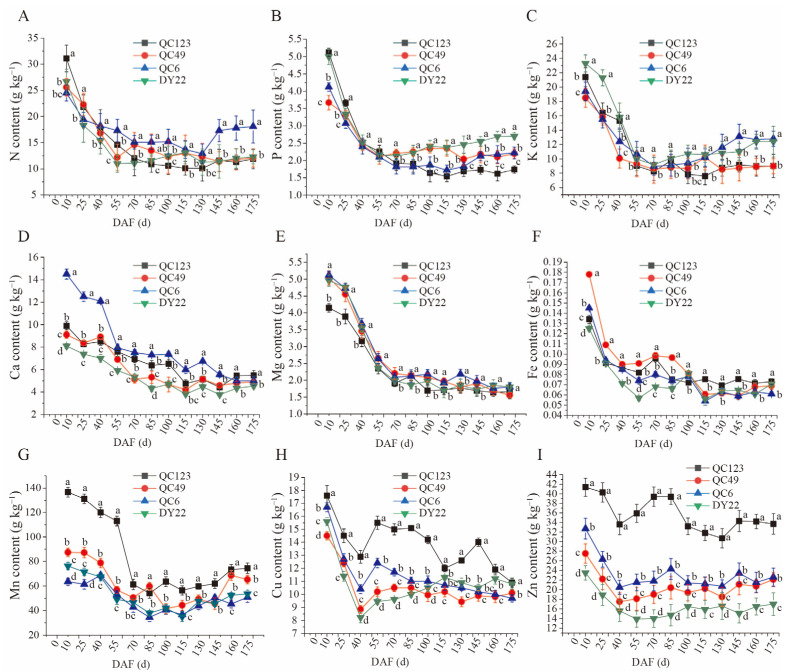
The dynamic of mineral elements in four elites’ fruit during the development. (**A**): N content, (**B**): P content, (**C**): K content, (**D**): Ca content, (**E**): Mg content, (**F**): Fe content, (**G**): Mn content, (**H**): Cu content, (**I**): Zn content. Different letters indicate significant differences (*p* < 0.05) within the different lines.

The fruit nutrient of N contents in the four elites declined drastically from 0 to 70 DAF, then maintained a relatively stable stage of 10~14 g kg^−1^ from 70~130 DAF, while QC6 decreased slightly from 145~175 and kept the highest content of 18.2 g kg^−1^ until the last stage (Figure 6A). No significant difference among the other three elites in N content during the last three periods (Figure 6A) was noted. The same trends were found in P and K contents: DY22 maintained a higher content of P than the other three elites from 130 to 175 DAF, while QC6 and DY22 kept the highest content of K at the end of the fruit development stage (Figure 6B,C). For the macroelements in fruit, Ca and Mg decreased during the fruit development, and there were no significant differences at the end of the fruit development on 175 DAF (Figure 6D,E). Even though the microelements of Fe, Mn, Cu, and Zn decreased after flowering, QC123 maintained relatively higher contents than the other three elites (Figure 6F–I). Moreover, the microelement contents of Mn, Cu, and Zn of QC123 were higher during the initial time than other three elites. The Zn content in DY22 was lower than that of the other elites after flowering (Figure 6I).

## 4. Discussion

Providing a thorough and standardized depiction of *Idesia polycarpa* phenology holds significant importance in agricultural production. However, past research on *Idesia polycarpa* has concentrated on cultivation methodologies, nutritional compositions, and medicinal applications. Therefore, this study describe the phenological stages of *Idesia polycarpa* preliminarily. Our study of the characteristics and dynamic changes of leaves, shoots, and flowers will serve as a practical guide for field management, ultimately enhancing cultivation practices, yield, landscape esthetics, and overall industrial development (Figure 1, Figure 2 and Figure 3 and Table 2).

The leaf features, such as size, shape, texture, and amount of pubescence might be key characteristics for new varieties or hybrids. The leaves plays important roles in the plant’s yield because of their main photosynthesis organ and their agronomic, phyto-physicochemical and stress-resilient traits for crop improvement and breeding [23]. Leaf traits, such as leaf area, dry or fresh weight, leaf length and width, leaf shape index, and so on, are important for the plant growth and stress resistance of Chinese chestnut [24]. The shape and number of leaves are related to the photosynthesis ability, yield, water use efficiency, and even desirability to consumers [25,26,27]. Leaf morphology, including the size, shape, and structure, plays a crucial role in photosynthesis, transpiration, and gas exchange [28,29]. Moreover, leaf morphology is tightly linked to plant health, growth vigor conditions, and productivity in agricultural and horticultural practices [30,31]. Therefore, comprehensive leaf and shoot morphologies of *Idesia polycarpa* Maxim are essential for improved cultivation practices and fruit yield production. We determined the leaf, petiole, and shoot development parameters of *Idesia polycarpa* during growth time (Figure 2). It is worth noting that the shoot weight decreased slightly from 195 to 240 DAF, which might be the decrease in precipitation and the water loss from the shoot (Figure 2C). Our study unravels the intricate biological progress governing growth and development to shed light on the transition of *Idesia polycarpa* Maxim from infancy to adulthood.

The flower and inflorescence growth, development, and opening time are of paramount importance for the fruit, even the oil production of *Idesia polycarpa*. In addition, to identify the sex of *Idesia polycarpa* Maxim is important for the yield production, as only male trees can offer fruit for the oil production. The floral features, such as corolla types, corona attributes, and morphometric traits could be distinguished for various hoya species [32]. These features, including the amount of hairiness on the leaves and outer surface of the corolla, the noticeable column, and the size and form of the pollinarium could distinguish different hoya species [33,34]. In addition, a relatively large flower size or color changes indicate more nectar rewards to some extent [35,36]. In this study, we measured the flower characteristic of *Idesia polycarpa* to explore the role of types and size in the tree’s development (Figure 1 and Figure 3). Moreover, understanding the disorders of sex development contributes to identifying the male and female *Idesia polycarpa* trees as early as possible, which is beneficial for the farmers to obtain more fruit for oil production (Figure 3). The study of flowering and fruit also offers opportunities for flower thinning to improve the fruit quality and oil composition [37]. Study of the flower has also balanced the nutrition uptake between flower and fruit and has then reduced the numerous flower and fruit abscissions during development in apple, olive, and *Camellia oleifera* trees [37,38,39]. Therefore, understanding the process of transition to maturity and floral development can guide commercial practices for the production of floral biomass, nutrient accumulation, oil fatty acid composition, and fruit development.

*Idesia polycarpa* exhibits dioecious characteristics, typically with separate male and female trees. Some studies reported the *Idesia polycarpa* individual tree had male flowers only, female flowers only, and bisexual flowers. In the field surveys, we found that the bisexual flowers were borne on the same inflorescence as the male flowers, and most female flowers were located at the top site, while male flowers were located at the button site (Figure 1 and Figure 3). We also observed in the bisexual individual trees that most inflorescences were male, and only a small number of inflorescences had bisexual flowers (Figure 1 and Figure 3). Moreover, the male flowers account for the majority in the bisexual inflorescences. However, occasional instances of sex conversion have been observed in *Idesia polycarpa* individuals [8]. In the middle of May, most male flowers were dead due to the abortion of the ovule at an early stage of development. After two years (the year from 2023 to 2024) of field observation statistics, most of the bisexual flowers were aborted, and only a few of the bisexual flowers could be pollinated normally and produce fruit.

By evaluating the fatty acids content and composition, the results can be important in distinguishing and classifying cultivars in pine nuts and walnut [21,40]. The fatty acids were different among different walnut cultivars and were affected by different climate zones [21]. Previous studies have shown that the content of linolenic acid in *Idesia polycarpa* was higher than that of other woody oils and that linolenic acid plays an important role in the development of fetuses and infants and as a cure for hypertension and diabetes [7,41,42,43]. This study also showed different contents in the fatty acids composition in four elites of *Idesia polycarpa*, which offered the possibility for cultivar classification (Figure 4 and Figure 5).

The number of fruits and the size and color influence the quality, including yield, nutrient accumulation, and oil fatty composition [37,44,45]. Screening the high content and large proportion of phospholipids and glycerolipids in mature pecan kernels provides a theoretical basis for the processing and utilization of plant and edible oils [46]. The *Idesia polycarpa* pulp oil was abundant in unsaturated fatty acids, which were composed of linoleic acid, oleic acid, palmitoleic acid [12]. In this study, it is interesting that the content of palmitoleic acid (C16:1) increased in fruit during the development of the four elites (Figure 6B). C16:1 might be a very unique fatty acid species in Idesia polycarpa, and we will continue to study its accumulation pattern and function. Thinning to obtain a reasonable number of flowers and fruits could reduce competition because of the simultaneous growth of *Idesia polycarpa*, which could maintain the growth potential and the number of flowers and fruits in the next year. We analyzed the fruit and fatty acid content of four elites to provide an theory for the fruit development and oil accumulation of *Idesia polycarpa* (Figure 3, Figure 4 and Figure 5).

The content of mineral elements in fruit can provide suggestions for fertilization. A lack of mineral elements may cause slower growth and development of plants and even lower production. Nitrogen plays a crucial role in synthesizing nucleic and amino acids and producing proteins and is a key component of vitamins, enzymes, and chlorophyll [47]. Some studies have proved that nitrogen is the limiting nutrient in the yield of pecans [48,49,50]. The application of calcium and boron fertilizers could reduce the pear cracking rate and improve the fruit quality [51]. The mineral content of N, P, K, Mg, Ca in leaves showed statistical variability among different guava (*Psidium guajava* L.) genotypes [52]. In addition, the minerals Ca, Na, Fe, Cu, P, and Zn in walnuts showed moderate associations with cultivars [21]. Our study showed that the macroelement and microelement contents were different among the four cultivates of *Idesia polycarpa*; however, they showed relatively similar trends during the fruit development (Figure 6). However, the mineral elements in fruits are closely related to the mineral elements in soil; we will further study the relationship between fruit and soil.

## 5. Conclusions

In summary, the integrative investigation of leaves, shoots, flowers, and the phenological period during the whole development of *Idesia polycarpa* revealed a basis for development characteristics which will be beneficial in guiding the production of *Idesia polycarpa* in terms of directing fertilization, pruning, weed and pest management, and disease control, as well as regulating flower and fruit production. In addition, the study of fatty acids showed that linoleic acid was the predominant fatty acid of fruit oil and that it showed differences among different elites. Moreover, the results of mineral content changes in fruit offers a basis theory for fertilization and soil management. Taken together, our findings show that *Idesia polycarpa* will be a promising choice of woody oil plant for edible oil production and functional components in the food industry in the future.

## Figures and Tables

**Table 1:** Examined morphological characteristics of *Idesia polycarpa*.

No.	Characters	Remarks
1	Flower types	Identification of the male, female, and bisexual flowers; the diameter of different flower types; and the diameter of male and female flowers during flowering time
2	Leaf length, width, and weight	Measured from the leaf in the middle of the shoot
3	Petiole length and weight	Measured the petiole indexes from the leaf in the middle of the shoot
4	Shoot length and weight	Measured the indexes of one-year-old shoot
5	Inflorescence indicators	Morphology and identification of the male, female, and bisexual inflorescence; the flowering sequencing; the length, width, and single weight of inflorescence on four-year-old trees; the inflorescence types; the length and width of inflorescence; the length/width ratio of 5- and 7-year-old trees
6	Oil content of fruit	Measured the total fatty acid content and composition of fatty acids of fruit during development
7	Fruit nutrients and mineral elements	Measured the nitrogen, phosphorus, potassium, calcium, magnesium, iron, manganese, copper, and zinc content of fruit during development

**Table 2:** The inflorescence determination of male and female between 5- and 7-year-old trees. Different letters indicate significant differences (*p* < 0.05) within the different lines.

	Inflorescence Types	The Length of Inflorescence	The Width of Inflorescence	Flowering Density	Length/Width Ratio
5-year-old	female flower	18.25 ± 6.39 b	8.33 ± 1.88 c	0.85 ± 0.14 a	2.22 ± 0.68 b
	male flower	17.98 ± 3.99 c	8.66 ± 2.06 b	0.77 ± 0.21 b	2.11 ± 0.32 c
7-year-old	female flower	21.08 ± 5.71 a	8.29 ± 2.4 c	0.8 ± 0.17 a	2.62 ± 0.57 a
	male flower	18.48 ± 7.46 b	9.13 ± 2.02 a	0.69 ± 0.27 c	2.04 ± 0.78 d

**Table 3:** The phenological period of *Idesia polycarpa*.

**Develop Periods**	**Bud Burst**	**Leaf Expansion**	**Inflorescence Growth**	**Initial Flowering**	**Full Flowering**	**Flower Decline**
Date (month)	18 March~28 March	20 March~30 April	25 March~25 April	20 April	20 April~1 May	1 May~5 May
**Develop Periods**	**Initial Fruiting**	**Fruit Enlargement**	**Fruit Color Change**	**Fruit Ripening**	**Post-Ripening**	**Leaf Fall**
Date (month)	30 April~5 May	5 May~1 June	10 August~5 October	14 October~27 October	27 October~15 January (next year)	25 October~ 25 November

## Data Availability

Data is contained within the article.

## References

[1] Kim S., Sung S., Choi S., Chung Y., Kim J. (2005). Idesolide: A new spiro compound from *Idesia polycarpa*. Org. Lett..

[2] Li T., Li F., Mei L., Li N., Yao M., Tang L. (2019). Transcriptome analysis of *Idesia polycarpa* Maxim. var vestita Diels flowers during sex differentiation. J. For. Res..

[3] Fan R., Li L., Cai G., Ye J., Liu M., Wang S., Li Z. (2019). Molecular cloning and function analysis of *FAD2* gene in *Idesia polycarpa*. Phytochemistry.

[4] Li N., He L., Huang L., Li T., Wang T., Tang L. (2020). Amelioration by *Idesia polycarpa* Maxim. var. vestita Diels. of oleic acid-induced nonalcoholic fatty liver in HepG2 cells through antioxidant and modulation of lipid metabolism. Oxidative Med. Cell. Longev..

[5] Wang Q., Wang Y., Yao S., Song H. (2012). Biodegradable lubricant from *Idesia polycarpa* Maxim. var. vestita Diels oil. Adv. Mater. Res..

[6] Yang F., Su Y., Li X., Zhang Q., Sun R. (2009). Preparation of biodiesel from *Idesia polycarpa* var. vestita fruit oil. Ind. Crops Prod..

[7] Hwang J., Moon S., Lee C., Byun M., Kim A., Sung M., Park H., Hwang E., Sung S., Hong J. (2012). Idesolide inhibits the adipogenic differentiation of mesenchymal cells through the suppression of nitric oxide production. Eur. J. Pharmacol..

[8] Wang H., Rana S., Li Z., Geng X., Wang Y., Cai Q., Li S., Liu Z. (2022). Morphological and anatomical changes during floral bud development of the trioecious *Idesia polycarpa* Maxim. Braz. J. Bot..

[9] Sohel R., Zhen L. (2021). Study on the pattern of vegetative growth in young dioecious trees of *Idesia polycarpa* maxim. Trees.

[10] Zhang C., Zhao X., Gao L., Gadow K. (2009). Gender, neighboring competition and habitat effects on the stem growth in dioecious *Fraxinus* mandshurica trees in a northern temperate forest. Ann. Forest Sci..

[11] Xiang X., Wen L., Wang Z., Yang G., Mao J., An X., Kan J. (2022). A comprehensive study on physicochemical properties, bioactive compounds, and emulsified lipid digestion characteristics of *Idesia polycarpa* var. vestita Diels fruits oil. Food Chem..

[12] Zhang W., Zhao C., Karrar E., Du M., Jin Q., Wang X. (2023). Analysis of chemical composition and antioxidant activity of *Idesia polycarpa* pulp oil from five regions in China. Foods.

[13] Hou K., Yang X., Bao M., Chen F., Tian H., Yang L. (2018). Composition, characteristics and antioxidant activities of fruit oils from *Idesia polycarpa* using homogenate-circulating ultrasound-assisted aqueous enzymatic extraction. Ind. Crops Prod..

[14] Zhou D., Zhou X., Shi Q., Pan J., Zhan H., Ge F. (2022). High-pressure supercritical carbon dioxide extraction of *Idesia polycarpa* oil: Evaluation the influence of process parameters on the extraction yield and oil quality. Ind. Crops Prod..

[15] Kong M., Kang J., Han C., Gu Y., Siddique H., Li F. (2020). Nitrogen, phosphorus, and potassium resorption responses of alfalfa to increasing soil water and P availability in a semi-arid environment. Agronomy.

[16] Ma Y., Zhang S., Wu Z., Sun W. (2022). Metabolic variations in brown rice fertilised with different levels of nitrogen. Foods.

[17] (2008). Vernier, Dial and Digital Display Calipers.

[18] AOCS (2011). Official Methods and Recommended Practices of the American Oil Chemist’s Society.

[19] Shi L., Mao J., Zheng L., Zhao C., Jin Q., Wang X. (2018). Chemical characterization and free radical scavenging capacity of oils obtained from *Torreya grandis* Fort. ex. Lindl. and *Torreya grandis* Fort. var. Merrillii: A comparative study using chemometrics. Ind. Crops Prod..

[20] (2000). Animal and Vegetable Fats and Oils—Preparation of Methyl Esters of Fatty Acids.

[21] Wu S., Ni Z., Wang R., Zhao B., Han Y., Zheng Y., Liu F., Gong Y., Tang F., Liu Y. (2020). The effects of cultivar and climate zone on phytochemical components of walnut (*Juglans regia* L.). Food Energy Secur..

[22] Bao S. (2000). Soil Agrochemical Analysis.

[23] Amin G., Kong K., Sharmin R., Kong J., Bhat J., Zhao T. (2019). Characterization and apid gene-mapping of leaf lesionmimicphenotypeof spl-1mutantinsoybean (*Glycine max*(L.)Merr.). Int. J. Mol. Sci..

[24] Jiang X., Wang Y., Lai J., Wu J., Wu C., Hu W., Wu X., Gong B. (2023). The construction of a high-density genetic map for the interspecific cross of *Castanea mollissima* × *C. henryi* and the identification of QTLs for leaf traits. Forests.

[25] Du M., Xiong M., Chang Y., Liu Z., Wang R., Lin X., Zhou Z., Lu M., Liu C., Liu E. (2022). Mining candidate genes and favorable haplotypes for flag leaf shape in Rice (*Oryza sativa* L.) based on a genome-wide association study. Agronomy.

[26] Bu S., Zhan P., Huang L., Tang J., Chen L., Zhu H., Liu Z., Meng L., Liu G., Wang S. (2022). Identification, interaction, expression, and function of QTLs on leaf numbers with single-segment substitution lines in Rice. Agronomy.

[27] Yi Q., López-Malvar A., Álvarez-Iglesias L., Romay M., Revilla P. (2023). Genome-wide association analysis identified newly natural variation for photosynthesis-related traits in a large maize panel. Agronomy.

[28] Jin D., Henry P., Shan J., Chen J. (2021). Identification of phenotypic characteristics in three chemotype categories in the genus *Cannabis*. HortScience.

[29] Byrne M. (2022). Plant development: Elementary changes determine leaf shape complexity. Curr. Biol..

[30] Carlson C., Stack G., Jiang Y., Taskıran B., Cala A., Toth J., Philippe G., Rose J., Smart C., Smart L. (2021). Morphometric relationships and their contribution to biomass and cannabinoid yield in hybrids of hemp (*Cannabis sativa*). J. Exp. Bot..

[31] Manica B., Teresa G., Daniel V., Oriane H., Daniel H. (2023). Intra-leaf modeling of *Cannabis* leaflet shape produces synthetic leaves that predict genetic and developmental identities. bioRxiv.

[32] Calpo J., Tiama N. (2023). Morphological characterization of hybrids derived from the pollination of *Hoya deleoniorum*. Biol. Life Sci. Forum..

[33] Aurigue F., Sahagun J., Suarez W. (2013). *Hoya cutis-porcelana* (Apocynaceae): A new species from samar and biliran islands, Philippines. J. Nat. Stud..

[34] Aurigue F., Cabactulan D., Pimentel R., Sahagun J. (2018). A remarkable new *Hoya* (Apocynaceae: Asclepiadoideae) from Mindanao, Philippines. Avonia.

[35] Sissi L., Nadine N., Yuval S. (2023). Flower size as an honest signal in Royal Irises (*Iris* Section *Oncocyclus*, Iridaceae). Plants.

[36] Tavares D., Freitas L., Gaglianone M. (2016). Nectar volume is positively correlated with flower size in hummingbird-visited flowers in the Brazilian Atlantic forest. J. Trop. Ecol..

[37] Ye T., Liu X., Liang X., Zhu X., Bai Q., Su S. (2022). Flower thinning improves fruit quality and oil composition in *Camellia oleifera* Abel. Horticulturae.

[38] Peifer L., Ottnad S., Kunz A., Damerow L., Blanke M. (2018). Effect of non-chemical crop load regulation on apple fruit quality, assessed by the DA-Meter. Sci. Hortic..

[39] Haouari A., Van Labeke M., Steppe K., Mariem F., Braham M., Chaieb M. (2013). Fruit thinning affects photosynthetic activity, carbohydrate levels, and shoot and fruit development of olive trees grown under semiarid conditions. Funct. Plant Biol..

[40] Mikkelsen A., Jessen F., Ballin N. (2014). Species determination of pine nuts in commercial samples causing pine nut syndrome. Food Control.

[41] Bowen R., Clandinin M. (2005). Maternal dietary 22: 6n-3 is more effective than 18: 3n-3 in increasing the 22: 6n-3 content in phospholipids of glial cells from neonatal rat brain. Br. J. Nutr..

[42] Eguchi K., Manabe I., Oishi-Tanaka Y., Ohsugi M., Kono N., Ogata F., Yagi N., Ohto U., Kimoto M., Miyake K. (2012). Saturated fatty acid and TLR signaling link β cell dysfunction and islet inflammation. Cell Metab..

[43] Marangoni F., Agostoni C., Borghi C., Catapano A.L., Cena H., Ghiselli A., La Vecchia C., Lercker G., Manzato E., Pirillo A. (2020). Dietary linoleic acid and human health: Focus on cardiovascular and cardiometabolic effects. Atherosclerosis.

[44] Castro D., ÁLvarez N., Gabriel P., Micheloud N., Buyatti M., Gariglio N. (2015). Crop loading studies on *‘Caricia’* and *‘Eva’* apples grown in a mild winter area. Sci. Agric..

[45] Celton J., Kelner J., Martinez S., Bechti A., Touhami A., James M., Durel C., Laurens F., Costes E. (2014). Fruit self-thinning: A trait to consider for genetic improvement of apple tree. PLoS ONE..

[46] Wang F., Zhao Z., Hu T., Zhou C. (2023). Identification of fatty acid components and key genes for synthesis during the development of pecan fruit. Horticulturae.

[47] Hawkesford M., Horst W., Kichey T., Lambers H., Schjoerring J., Møller I.S., White P., Marschner P. (2012). Functions of macronutrients. Marschner’s Mineral Nutrition of Higher Plants.

[48] Wells M.L., Harrison K.A. (2010). Cultural Management of Commercial Pecan Orchards.

[49] Ren W., Zhang L., Maness N., Wang X., Tang M., Xu T. (2023). Changes in the diversity of pecan (*Carya illinoinensis*) rhizosphere microbial community with different nitrogen fertilization, a case study in Oklahoma pecan orchard. Sci. Hortic..

[50] Kim T., Mills H.A., Wetzstein H.Y. (2002). Studies on effects of nitrogen form on growth, development, and nutrient uptake in pecan. J. Plant Nutr..

[51] Seo H.J., Sawant S.S., Lee B., Kim K., Song J., Choi E.D. (2024). Mechanisms driving fruit cracking in ‘Sinhwa’ pears (*Pyrus pyrifolia* Nakai) and effect of foliar fertilizer application on fruit quality. Sci. Hortic..

[52] Qin J., Shao X., Zhang S., Chen X., Lai D., Xiao W., Zhuang Q., Kuang S. (2023). Evaluation of bioactive compounds, antioxidant capacity and mineral elements in the leaves of guava (*Psidium guajava* L.) genotypes from China. Sci. Hortic..

